# Coefficients optimization of the GLASS broadband emissivity based on FTIR and MODIS data over the Taklimakan Desert

**DOI:** 10.1038/s41598-019-54982-6

**Published:** 2019-12-05

**Authors:** Aynigar Yalkun, Ali Mamtimin, Suhong Liu, Fan Yang, Qing He, Feifei Qi, Yongqiang Liu

**Affiliations:** 1 College of Resources & Environmental Sciences, Xinjiang University, Urumqi, 830046 China; 2 Institute of Desert Meteorology, China Meteorological Administration, Urumqi, 830002 China; 3 Xinjiang Meteorological Bureau, Urumqi, 830002 China

**Keywords:** Climate and Earth system modelling, Environmental impact

## Abstract

In this paper, the Taklimakan Desert land surface emissivity measured by portable Fourier Transform thermal InfraRed spectroscopy (FTIR) was used to re-estimate Global LAnd Surface Satellite (GLASS) BroadBand Emissivity (BBE) and Moderate Resolution Imaging Spectroradiometer (MODIS) BBE optimal coefficients equations. In addition, the revised BBE equations of both GLASS and MODIS with the optimized coefficients were obtained. Comparing the FTIR and MODIS BBE data with the values retrieved before and after the revised GLASS BBE equation, it was found that the revised GLASS BBE equation is more accurate than the original one. First, according to the error analysis with FTIR data, the value of R^2^ is increased from 0.4 to 0.9, and the Root Mean Square Error (RMSE) and Bias are reduced by 1 and 3 magnitude orders, respectively. Second, with MODIS BBE data, the value of R^2^ is increased from 0.6 to 0.9, the RMSE and Bias are reduced by 1 and 2 magnitude orders, respectively. Finally, the Taklimakan Desert BBE was calculated using the revised GLASS BBE equation. The results showed that the BBE values are between 0.890 and 0.920 in the desert center, between 0.920 and 0.950 in the sparse vegetation areas, and between 0.950 and 0.980 in the oasis edges.

## Introduction

The land surface broadband emissivity (BBE) is a key variable for estimating land surface longwave net radiation, which is a surface radiation budget component and an important parameter in climate, weather, and hydrological models^1–6^. It is the most effective and accurate method to calculate the emissivity based on the obtained spectral data of the broadband surface emissivity.

Broadband emissivity spectra can be accurately measured by using Fourier transform infrared spectrometer (FTIR)^7–11^. Liu Yongqiang *et al*.^12^ and Liu *et al*.^13^ used FTIR to study the surface emissivity in the hinterland of Taklimakan Desert for the first time. Additional, thermal infrared remote sensing method is an effective method for getting the emissivity of the area. But thermal infrared remote sensing method can only obtain specific narrow-band emissivity and cannot represent the broadband emissivity^14^. However, due to the lack of reliable observations, a constant emissivity value or very simple parameterized models of the emissivity are currently used in Land Surface Models (LSM) and General Circulation Models (GCM)^3,15^. For example, the land model version 2 by the National Center for Atmospheric Research, USA calculates the canopy emissivity from the leaf area index and sets the soil and snow emissivities as 0.96 and 0.97, respectively^16^.

A sensitivity study of the simulated energy balance to the changes in emissivity in North Africa and the Arabian Peninsula indicated that a decrease of the soil BBE by 0.1 will cause the increases of the ground temperature and air temperature by an average of approximately 1.1 °C and 0.8 °C, respectively, and the decreases in the net and upward longwave radiation by approximately 6.6 W·m^−2^ and 8.1 W·m^−2^, respectively^15^. Jin and Liang^3^ also revealed the contributions of the BBE in improving the climate models simulation results.

Therefore, when estimating the broadband surface emissivity, most of the researchers would first select the emissivity remotely sensed data of the thermal infrared band to establish the broadband emissivity estimation model. Ogawa *et al*.^17^ determined the optimal broadband window for estimating longwave net radiation in the range from 8 μm to 13.5 μm, using the narrowband emissivity from MODIS and ASTER (Advanced Space borne Thermal Emission and Reflection Radiometer) to build the broadband emissivity model. They estimated the broadband land surface emissivity in the Sahara Desert and a cross-check was conducted.

Wang *et al*.^18^ established an emissivity estimation model for the band range from 8 μm to 13.5 μm using five thermal infrared bands linear combination from ASTER to estimate the longwave net radiation and analyzed the correlation between the emissivity and soil moisture, but did not verify the estimated emissivity accuracy. Wang *et al*.^19^ used the MODIS thermal infrared band data to estimate the surface emissivity of the window from 3 μm to 14 μm and verified the results using the MODIS emissivity library.

Tang *et al*.^20^ selected two broadband windows from 3 μm to14 μm and from 3 μm to ∞ μm respectively, then established broadband radiance estimation by using MODIS UCSB (University of California, Santa Barbara) emissivity library and ASTER spectral library (JHU & JPL spectral library) data. The model still used the spectral library data to validate the model and its estimation accuracy about soil, vegetation, water bodies, ice, and snow. Ogawa *et al*.^21^ also used MODIS data from the 7th band, but without using the ground observation data the applicability of these estimation equations in local areas is limited.

In view of the shortcomings of the above methods in estimating the surface BBE, this study used the FTIR measured data to optimize the coefficients in the GLASS BBE equation to calculate the BBE data in the Taklimakan Desert. Through the comparison and analysis between FTIR data with MODIS and GLASS BBE products data. In addition, the consistency of the GLASS BBE products on the spatial scale was evaluated. The GLASS and MODIS BBE distribution in the Taklimakan Desert was analyzed and FTIR data was used directly to verify and evaluate the GLASS BBE accuracy.

## Results

### Estimation equation

Li *et al*.^22^ pointed out that MODIS emissivity product data accuracy is preferably when FTIR measurement data is used and the coefficients of the formula (9) are fitted by using 10 measurement values as follows (Eq. 1):

1 $$y=0.0675{\varepsilon }_{29}+0.1326{\varepsilon }_{31}+0.7842{\varepsilon }_{32}-0.1206{\rho }_{7}+0.0071$$

**Figure 1:**
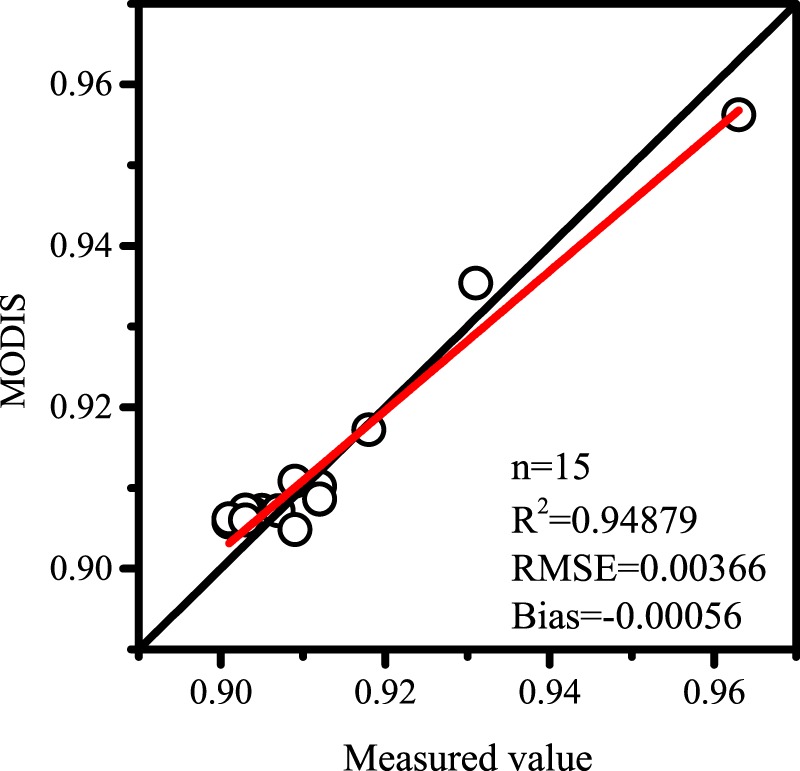
Emissivity comparison between FTIR observed values and MODIS estimated values. The graph was generated with OriginPro Portable 9.0.0. (https://www.originlab.com/).

The rest 15 values were used to verify the results (Fig. 1). It can be shown that The fitting result of the MODIS BBE estimation equation obtained by using FTIR data is better than the original, and R^2^ reaches 0.95. Therefore, the value of MODIS fitted by Eq. (1) can be used to verify GLASS BBE.

### Comparative verification

**Figure 2:**
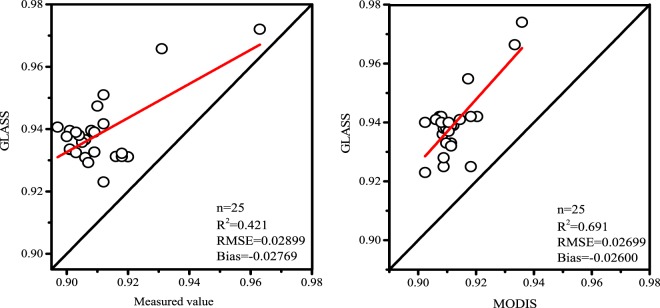
Comparison between FTIR observation (left) and MODIS estimation (right) with GLASS BBE. The graph was generated with OriginPro Portable 9.0.0. (https://www.originlab.com/).

First, extract the image value from the same position as the FTIR measured point from GLASS, were compared with MODIS emissivity products and the FTIR values, respectively. The results (Fig. 2) show that the GLASS products correlation with either the FTIR or MODIS is relatively low before the GLASS products are revised. The R^2^ with the FTIR was 0.421, and RMSE and Bias were 0.02899 and −0.02769, respectively. The correlation with MODIS was better than the correlation with FTIR, but its RMSE and Bias were both generally high. Therefore, GLASS product gives high emissivity values and its accuracy in describing the desert needs to be improved.

We fitted the GLASS data by randomly selecting 10 figures from the measurement data and using the multiple regression method to obtain the equation coefficient (10) as follows (Eq. 2):

2 $${\varepsilon }_{8-13.5}=0.235{\alpha }_{1}-0.724{\alpha }_{2}-0.325{\alpha }_{3}+0.231{\alpha }_{4}+0.313{\alpha }_{5}+0.757{\alpha }_{6}-0.7126{\alpha }_{7}+0.964$$

**Figure 3:**
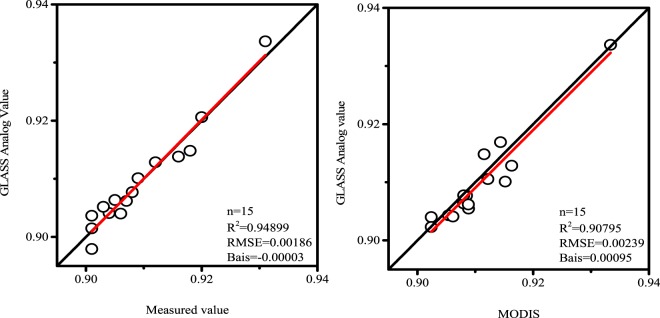
Comparison between FTIR observation (left) and MODIS estimation (right) with modified GLASS BBE estimations. The graph was generated with OriginPro Portable 9.0.0. (https://www.originlab.com/).

The rest 15 figures were taken into the fitted Eq. (2) and the result was compared with the FTIR measurement data and MODIS data respectively as shown in Fig. 3.

It is shown in Fig. 3 that for the revised GLASS data, its R^2^ with FTIR data has changed from 0.421 to 0.948, and its R^2^ with MODIS has changed from 0.691 to 0.907. The corresponding RMSE was reduced by a magnitude order and the Bias was also significantly reduced.

Since MOD43A3 is the albedo data, the value in the desert area and the vegetation area is opposite to the emissivity. Therefore, the equation value in the oasis zone around the Taklimakan Desert is not accurate enough. Therefore, in combination with the GLASS broadband emissivity algorithm and the value of the MODIS NDVI product, the algorithm for the entire region is re-fitted. So combined with re-fitting broadband emissivity of GLASS and the product of MODIS NDVI. Out of the equation for the entire Taklimakan Desert as follows (Eq. 3):

3 $$\begin{array}{c}{\varepsilon }_{8-13.5}=0.036\cdot NDVI+0.235{\alpha }_{1}-0.724{\alpha }_{2}-0.325{\alpha }_{3}+0.231{\alpha }_{4}+0.313{\alpha }_{5}\\ \,+0.757{\alpha }_{6}-0.7126{\alpha }_{7}+0.964\end{array}$$

### Emissivity distribution characteristics in the Taklimakan Desert

**Figure 4:**
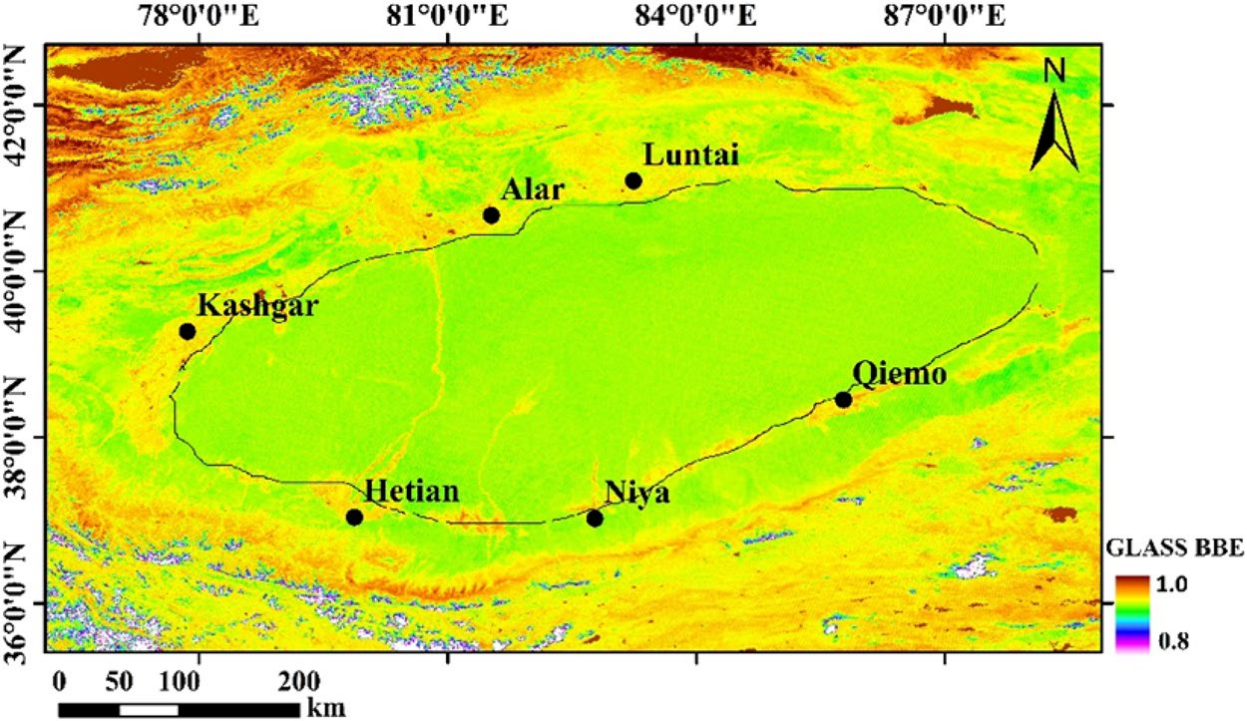
Distribution for the broadband emissivity over the Taklimakan desert (Lines are the boundaries between deserts and oases). The map was generated with ArcMap 10.0. (http://www.esri.com/en/arcgis/arcgis-for-desktop/).

The emissivity distribution in the Taklimakan Desert was conducted by selecting the bands from 1 to 7 of the black-air albedo from MODIS MOD43A2 product on September 27, 2014. The surface broadband emissivity was calculated using Eq. (3). The area is 1300 km × 500 km, including the entire Taklimakan Desert and the oases around the Tarim Basin and the southern Tianshan Mountains and the northern Kunlun Mountains (Fig. 4). It can be seen that the broadband emissivity value from the Taklimakan Desert region was centered on the range between 0.89 and 0.92, covering the majority of the area distribution. The values in the regions with sparse vegetation were between 0.92 and 0.95, and the values for the oases near the edge of the desert, such as Alar, Qiemo, Luntai, Kashgar oasis, were between 0.95 and 0.98.

## Conclusion and Discussion

We used the FTIR measured data to establish a multivariate linear regression equation for GLASS BBE products and to establish the equation with MODIS bands 7, 29, 31, and 32. The accuracy of the modified GLASS BBE equation is significantly improved in the Taklimakan Desert and some results are as follows:

First, the GLASS emissivity products correlation analysis was carried out by using the data measured by FTIR, the R^2^ of the measured value is 0.421, RMSE and Bias are 0.029 and −0.02769, respectively. For MODIS BBE, the R^2^ is 0.691, the RMSE and Bias are 0.0268 and −0.026, respectively, and the GLASS data were higher than FTIR and MODIS data.

Second, using the FTIR data to perform multivariate linear regression on the calculation formula of GLASS emissivity products, the correlation between the calculated new values and the values not participating in the regression was obtained, and the obtained R^2^ is significantly improved, which was increased by 0.528, RMSE and Bias were reduced by 0.01713 and 0.02766, respectively. For MODIS data correlation analysis, the R^2^ was also increased by 0.217, RMSE and Bias were decreased by 0.024 and 0.02505, respectively, and the results were significantly improved.

Finally, using the calculated regression equation to perform the band calculation on the MODIS43A3 image, it can be seen that the surface emissivity value reached the highest of 0.95 near the oasis, and was in the range between 0.91 and 0.95 in the oasis and desert transition zone, in the range between 0.88 and 0.91 in the rest of the desert. The value in the desert hinterland area was less than 0.88.

Although the estimation method proposed in this paper achieved a reasonable broad-band surface emissivity, it was only validated and analyzed in the Taklimakan Desert area, and it requires more extensive verification if it is applied in other desert areas, or in other vegetation coverage areas. Its applicability in wet areas also remains to be further studied.

### Study area and data

#### Study area

**Figure 5:**
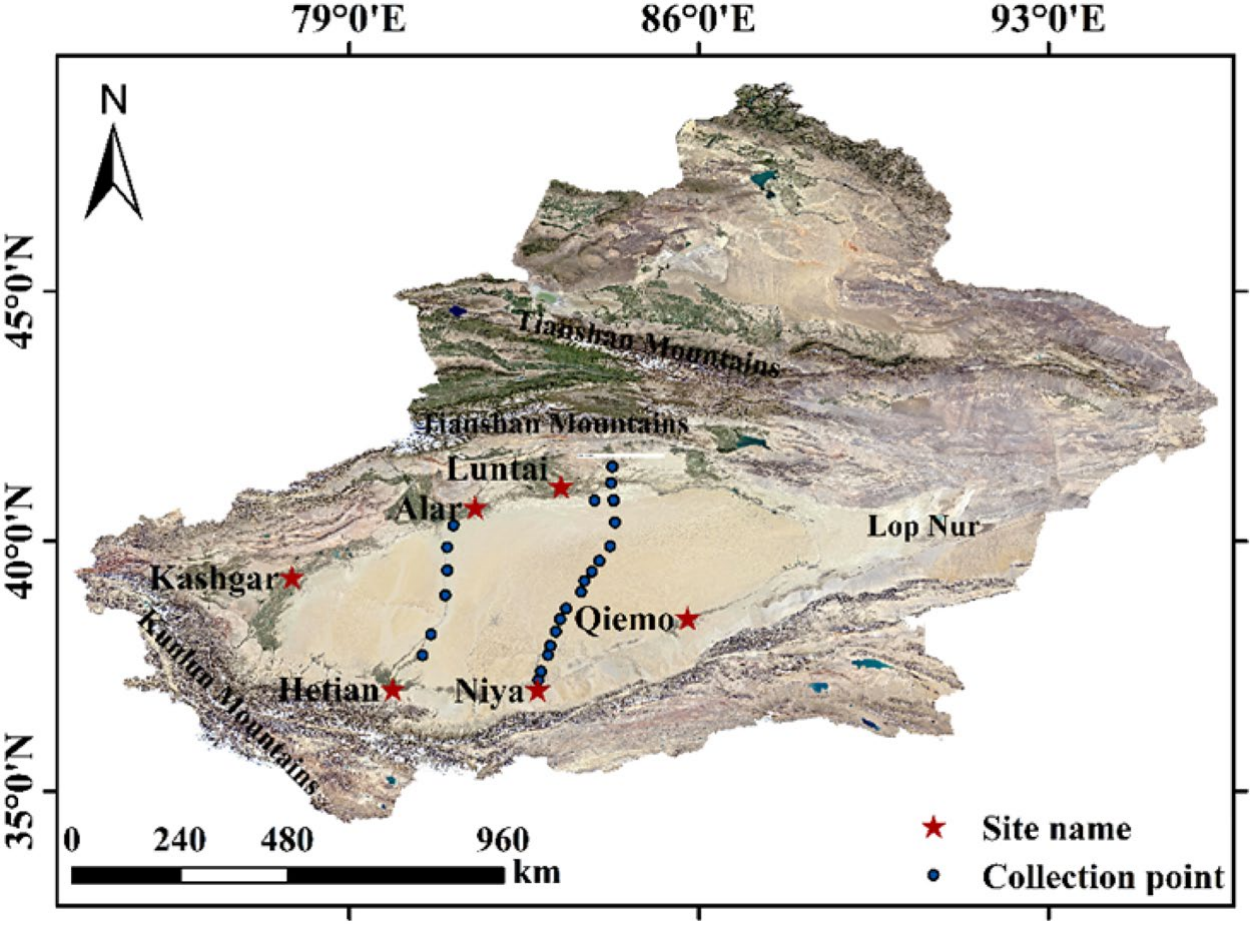
FTIR observed sites along two desert highways. The map is generated with ArcMap Version 10.0(Image download from Google Earth) (http://www.esri.com/en/arcgis/arcgis-for-desktop/).

The Taklimakan Desert is located in the middle of the Tarim Basin in Xinjiang of China. It is adjacent to the Tomur Mountains and the Pamirs in the west, the Kunlun Mountains and the Altun Mountains in the south, the Tianshan Mountains in the north and the Lop Nur in the east. It is about 1070 km long from east to west, 420 km wide from north to south, with an area of 33.76 × 10^4^ km^2^ and an altitude in the range from 800 to 1300 m. The terrain is high in the west and low in the east. It is the second-largest mobile desert in the world^23–26^. Desert soil type is single, mainly being silt, of which the most is fine sand, accounting for 43.8% to 75.5% of sediment transport, and a small amount of feldspar and muscovite^12^. According to the historical observation data of the Tazhong Meteorological Station in Xinjiang (39°00′N, 83°40′E, altitude 1099.3 m) from 1996 to 2014, the highest temperature in the region was up to 45.6 °C, the lowest temperature was −32.7 °C. The annual average wind speed was 2.5 m·s^−1^, and the instantaneous maximum wind speed was 24.0 m·s^−1^ (Fig. 5)^27^.

### Data

#### FTIR observation data

The field observation of the broadband emissivity in the Taklimakan Desert was conducted by using a portable Fourier transform thermal infrared spectroscopy (FTIR). The observed sites were picked up along two desert highways which cross through the desert from north to south (one is from Luntai to Niya, another from Alar to Hetian (Table 1). The soil composition and humidity change are small due to the single type of desert surface; thus, the suitable site was chosen every 50–100 km and the observed sites were increased in the oasis transition zone at the edge of the desert. In order to have effective observation data, the measurement was carried out on sunny days^22^. The field data collection was from Oct 16 to Oct 19, 2013, for the sites in the Luntai-Niya Desert Highway and from Sep 25 to Sep 27, 2014, for the sites in the Alar-Hetian Desert Highway. The data for a total of 25 sites were collected.

**Table 1:** Site place latitude and longitude coordinates.

Site place	Latitude	Longitude
Alar	40°22′–40°57′	80°30′–81°58′
Hetian	36°59′–37°14′	79°50′–79°56′
Luntai	41°05′–42°32′	83°38′–85°25′
Qiemo	35°40′–40°10′	83°25′–87°30′
Niya	35°20′–39°29′	83°38′–87°30′
Kashgar	35°20′–40°18′	73°20′–79°57′

#### MODIS data

MODIS data provides 36 discrete-band images between 0.4 to 14.4 μm, with high spectral resolution and time resolution. Global observations are available every 1 to 2 days for large scales. Significant advantages in large-scale monitoring of global environmental parameters such as temperature^28^. The MODIS data has 36 channels, of which the channels 1–19 and 26 are distributed in the visible and near-infrared bands, and the other 16 channels are distributed in the thermal infrared band from 3–14 μm. The thermal infrared band atmospheric window that obtains the most suitable surface emissivity is in the range from 8 to14 μm, which correspond to the bands from 29 to 32. Because the 30th band has strong ozone absorption, it cannot be utilized. Therefore, the thermal infrared wave points were chosen band 29 (8.400–8.700 μm), band 31 (10.780–11.280 μm) and band 32 (11.770–12.270 μm). In addition, the 7th band (2.105–2.155 μm) in the near-infrared spectra is the reflection of surface and clouds^29^ which can represent the surface soil properties^30^, and thus the band 7 has the highest correlation with the broadband emissivity in all MODIS reflection channels^27^. The soils and rocks surface reflectivity depends on their mineral composition and emissivity. In general, the quartz-rich (SiO_2_) sand has higher reflectivity and lower emissivity, but the mafic minerals with lower SiO_2_ content generally have lower reflectance^21^. The Taklimakan Desert is rich in quartz sand and has a high surface reflectivity^23,30^, therefore the 7th band was added to the study.

We used the bands 31and 32 from MOD11A1 which is a MODIS product about the temperature versus emissivity, the band 29 from MOD11B1 (with a resolution of 1000 m) and the band 7 reflectance data (with a resolution of 500 m) from MOD09GA which is the land secondary standard product. The acquired image data covered the entire Taklimakan Desert in the time period from Oct 16 to Oct 18, 2013, and from Sep 25 to Sep 27, 2014. The MODIS data were downloaded from the National Aeronautics and Space Administration (NASA) website at http://modis.gsfc.nasa.gov.

#### GLASS data

The GLASS emissivity is a BBE (covering wavelength range from 8 μm to 13.5 μm) product derived from AVHRR VNIR data and MODIS albedos by using newly developed algorithms^31,32^. GLASS emissivity has two parts: (i) the global eight-day 1 km land surface BBE retrieved from MODIS albedos from 2000 through 2010 and (ii) the global eight-day 5 km land surface BBE retrieved from AVHRR VNIR reflectance from 1981 through 1999.

In the algorithm used to generate GLASS BBE from MODIS albedos, the land surface was classified into five types according to Normalized Difference Vegetation Index (NDVI) threshold values, such as water, snow or ice, bare soil, vegetation, and transition zone. The overlapping areas of bare soil and transition zone or of transition zone and vegetation were noted. The BBEs of water and snow or ice were set as 0.985 based on a combination of BBE calculated from the emissivity spectrum in the ASTER spectral library and the MODIS USCB emissivity library, and BBE simulated through radiative transfer models^33^.

BBEs of bare soil, vegetated areas, and transition zones were formulated as the linear function of seven MODIS narrowband black-sky albedos. When NDVI was lower than 0.1 or higher than 0.2, the formula for bare soil or vegetated areas was used to calculate individual BBE values. In overlapping bare soil and transition zone areas (0.1 < NDVI ≤ 0.156), BBE was taken as the average of values calculated by using formulas for bare soil and transition zones. By contrast, BBE for areas of the overlapping transition zone and vegetated area (0.156 <NDVI< 0.2) was taken as the average values calculated by using formulas for transition zones and vegetated areas. BBE derived from MODIS albedos was validated by field measurements in the US and China, and the absolute difference was 0.02^31,34,35^.

## Methods

### Broadband emissivity measured by FTIR

The observation of surface-specific emissivity spectral data was collected by using a portable FTIR spectrometer (Model 102 F, Designs and Prototypes Ltd.) with the following parameters: a luminous flux of 0.016 cm^−2^·sr, an operating spectral range from 2 μm to16 μm, and a spectral resolution of 2–24 cm^−1^. The measurement results can reach a standard deviation of less than 1%^10^. The formula for calculating the surface-specific emissivity spectrum is as follows (Eq. 4):

4 $${\varepsilon }_{\lambda }=\frac{{L}_{\lambda }-L{D}_{\lambda }}{{B}_{\lambda }(T)-L{D}_{\lambda }}$$

where *ε*_*i*_ is the spectrum of the surface specific emissivity when the wavelength is *λ*, *L*_*λ*_ (cm^2^·sr) is the surface radiance of the wavelength *λ*, *B*_*λ*_ (*T*)(cm^2^·sr) is the black body radiance when the wavelength is *λ* and the surface temperature is *T*(K), downward radiation *D*_*λ*_ (cm^2^·sr) is measured using a calibrated diffuse gold plate^3,28^. Liu *et al*. and Liu Yongqiang *et al*. gave the operation process and observation results of the surface specific emissivity spectrum observation in the Taklimakan Desert^12,13^.

In the land-surface patterns and numerical prediction models, the surface-specific emissivity uses the broadband emissivity spectra average. The calculation method of the surface emissivity between the wavelength range from *λ*_1_ to *λ*_2_ and is shown as follows (Eq. 5)^36^.

5 $${\varepsilon }_{\lambda 1-\lambda 2}=\frac{{\int }_{{\lambda }_{2}}^{{\lambda }_{1}}{\varepsilon }_{\lambda }{B}_{\lambda }(T)d\lambda }{{\int }_{{\lambda }_{2}}^{{\lambda }_{1}}{B}_{\lambda }(T)d\lambda }$$

where *λ*_1_ and *λ*_2_ is the upper and lower wavelength range limits. Rather than what Ogawa *et al*.^17^ pointed out that the wavelength ranges from 8 μm to13.5 μm is the best window for estimating long-wave radiation when using FTIR to observe the surface emissivity spectrum, higher precision can be obtained in the range from 8 μm to 14 μm^13^. Therefore, the broadband emissivity is calculated using a wavelength range from 8 μm to 14 μm. Since *ε*_*λ*_ and *B*_*λ*_(*T*) are continuous functions, the integral equation is discretized as follows (Eq. 6):

6 $${\varepsilon }_{\lambda 1-\lambda 2}=\frac{{\sum }_{\lambda ={\lambda }_{1}}^{{\lambda }_{2}}{\varepsilon }_{\lambda }{B}_{\lambda }(T)\varDelta \lambda }{{\sum }_{\lambda ={\lambda }_{1}}^{{\lambda }_{2}}{B}_{\lambda }(T)\varDelta \lambda }$$

### Broadband emissivity measured by MODIS

The MODIS thermal infrared single-band surface emissivity is calculated as follows (Eq. 7)^37^.

7 $${\varepsilon }_{i}=\frac{{\int }_{{\lambda }_{i1}}^{{\lambda }_{i2}}{f}_{i}(\lambda ){\varepsilon }_{\lambda }{B}_{\lambda }(T)\varDelta d\lambda }{{\int }_{{\lambda }_{i1}}^{{\lambda }_{i2}}{f}_{i}(\lambda ){B}_{\lambda }(T)\varDelta d\lambda }$$

where *ε*_*i*_ is the MODIS thermal infrared single band emissivity, *f*_*i*_(*λ*) is the spectral response function of the band *i*. According to the Eqs. (2) and (4), the broadband emissivity can be calculated by a linear combination of the emissivity in different bands (Eq. 8)^21^.

8 $${\varepsilon }_{{\lambda }_{1}-{\lambda }_{2}}=\frac{{\sum }_{i=1}^{n}{\int }_{{\lambda }_{1}}^{{\lambda }_{(i+1)}}{\varepsilon }_{\lambda }{B}_{\lambda }(T)d\lambda }{{\int }_{{\lambda }_{1}}^{{\lambda }_{2}}B(T)d\lambda }\approx {\sum }_{i=1}^{n}{g}_{i}{\varepsilon }_{i}$$

where *g*_*i*_ is the combination coefficient, which is related to the black body thermal radiance when the temperature is *T*, and is independent of the single band-specific radiance and n is the number of bands. Thus, using the bands 29, 31 and 32 from MODIS data, the broadband emissivity estimation equation can be obtained by fitting the multiple linear regression equation. Since the band 7 of MODIS is highly correlated with the emissivity, it is added to the linear combination equation. Therefore, to calculate the broadband emissivity *ε*_8–13.5_, we have the following formula^22^ (Eq. 9).

9 $${\varepsilon }_{8-13.5}={\sum }_{i}{a}_{i}{\varepsilon }_{i}+b{\rho }_{7}+c$$

where, *i* = 29, 31, 32, *a*_*i*_, *b*, and *c* are the coefficients of the multiple regression equation, *ε*_*i*_ is the emissivity value for the bands 29, 31 and 32, *ρ*_7_ is the reflectance value of the band 7.

### Broadband emissivity estimated by GLASS

The global land surface is divided into five types as water body, ice/snow, bare soil, vegetation cover, and transition area. The emissivity estimation method is given respectively based on the type. (i) For water bodies, ice/snow pixels, the value is 0.985; (ii) For other surface types, using ASTER emissivity products, MODIS reflectance and albedo products as data sources, the empirical relationships were established between the narrowband black sky albedo and the BBEs of the bare soil, transition areas, and vegetation-covered areas. In addition, the broadband emissivity spectral range is from 8 μm to 13.5 μm and the algorithm for calculating the bare soil broadband emissivity is shown as follows (Eq. 10):

10 $$\bar{\varepsilon }=e+{\sum }_{i=1}^{7}{d}_{i}BS{A}_{i}$$

where, $\bar{\varepsilon }$ is broadband emissivity, *BSA*_*i*_ is a narrowband black sky albedo, e is a constant, d_i_ are bands.

For bare soil: NDVI <= 0.1, pixel emissivity from bare soil algorithm; vegetation cover: NDVI > = 0.2, two cases fully vegetated (NDVI > 0.461) and Mixed Scenario (0.2 < = NDVI < 0.461). The emissivity is derived from the vegetation cover algorithm; the transition area is subdivided into two parts: the bare soil transition zone and the vegetation transition zone. The soil transition zone: NDVI is 0.1–0.156, and the pixel emissivity is derived from the transition zone and the bare soil zone. Mean of the algorithm; vegetation transition zone: NDVI is 0.156–0.2, and the pixel emissivity is derived from the mean of the vegetation zone and transition zone algorithm, and the broadband surface emissivity of the vegetation area is expressed as (Eq. 11):

11 $$\bar{\varepsilon }={A}_{0}\cdot NDVI+{\sum }_{i=1}^{7}{f}_{i}\cdot BS{A}_{i}+h$$

where, *A*_0_ and *h* are constants, NDVI is the normalized vegetation index, *BSA*_*i*_ which are narrow-band black sky albedo, and *f*_*i*_ are the coefficient of albedo.

## References

[1] Cheng J, Liang S, Wang J, Li X (2010). A stepwise refining algorithm of temperature and emissivity separation for hyperspectral thermal infrared data, IEEE Trans. Geosci. Remote Sens.

[2] Jacob F (2004). Comparison of land surface emissivity and radiometric temperature derived from MODIS and ASTER sensors. Remote Sensing of Environment.

[3] Jin M, Liang S (2006). An Improved Land Surface Emissivity Parameter for Land Surface Models Using Global Remote Sensing Observations. Journal of Climate.

[4] Liang, S. Quantitative Remote Sensing of Land Surface//Quantitative remote sensing of land surfaces. *Wiley-Interscience*, 413–415. (2004)

[5] Liang S, Kustas W, Schaepman-Strub G, Li XW (2010). Impacts of Climate Change and Land Use Changes on Land Surface Radiation and Energy Budgets. IEEE Journal of Selected Topics in Applied Earth Observations & Remote Sensing.

[6] Péquignot E, Chédin A, Scott NA (2008). Infrared Continental Surface Emissivity Spectra Retrieved from AIRS Hyperspectral Sensor. Journal of Applied Meteorology & Climatology.

[7] Hanan NP (2005). Testing a model of CO_2_, water and energy exchange in Great Plains tallgrass prairie and wheat ecosystems. Agricultural & Forest Meteorology.

[8] Hook SJ, Kahle AB (1996). The micro fourier transform interferometer (micro FTIR)–A new field spectrometer for acquisition of infrared data of natural surfaces. Remote Sensing of Environment.

[9] Korb AR, Dybwad P, Wadsworth W, Salisbur JW (1996). Portable Fourier transform infrared spectroradiometer for field measurements of radiance and emissivity. Applied Optics.

[10] Korb AR, Salisbury JW, D’Aria DM (1999). Thermal-infrared remote sensing and Kirchhoff’s law: 2. Field measurements. Journal of Geophysical Research Solid Earth.

[11] Hori M (2006). *In-situ* measured spectral directional emissivity of snow and ice in the 8–14 μm atmospheric window. Remote Sensing of Environment.

[12] Liu YQ (2014). Characteristics of surface emissivity and distribution in the Taklimakan Desert. Desert and oasis weather.

[13] Liu YQ (2014). Estimation of the land surface emissivity in the hinterland of Taklimakan Desert. Journal of Mountain Science.

[14] Sobrino JA, Raissouni N, Li ZL (2001). A Comparative Study of Land Surface Emissivity Retrieval from NOAA Data. Remote Sensing of Environment.

[15] Zhou L., Dickinson R. E., Tian Y., Jin M., Ogawa K., Yu H., Schmugge T. (2003). A sensitivity study of climate and energy balance simulations with use of satellite-derived emissivity data over Northern Africa and the Arabian Peninsula. Journal of Geophysical Research: Atmospheres.

[16] Bonan GB (2002). The Land Surface Climatology of the NCAR Land Surface Model Coupled to the NCAR Community Climate Model. Journal of Climate.

[17] Ogawa K, Schmugge T, Rokugawa S (2008). Estimating Broadband Emissivity of Arid Regions and Its Seasonal Variations Using Thermal Infrared Remote Sensing. IEEE Transactions on Geoscience & Remote Sensing.

[18] Wang H, Xiao Q, Li H, Du Y, Liu Q (2015). Investigating the Impact of Soil Moisture on Thermal Infrared Emissivity] Using ASTER Data. IEEE Geoscience & Remote Sensing Letters.

[19] Wang, K. *et al*. Estimation of surface long wave radiation and broadband emissivity using Moderate Resolution Imaging Spectro radiometer (MODIS) land surface temperature/ /emissivity products. *Journal of Geophysical Research Atmospheres*, **110**(D11) (2005)

[20] Tang BH, Wu H, Li C, Li ZL (2011). Estimation of broadband surface emissivity from narrowband emissivities. Optics Express.

[21] Ogawa K, Schmugge T (2004). Mapping Surface Broadband Emissivity of the Sahara Desert Using ASTER and MODIS Data. Earth Interactions.

[22] Li HQ (2017). Estimating the Surface Broadband Emissivity of Deserts in Xinjiang base on MODIS and FTIR Data. Journal of Desert Research.

[23] Peng YM (2018). terland of the Taklimakan Desert[J]. Desert & Oasis Meteorology, 2018.Desert and Oasis Meteorology.

[24] Peng, Y., Wang, S., Xiao, G., He, Q. & Liu, X. C. Impact Factors of Atmospheric Aerosol Scattering Coefficient in the Tazhong Area of the Taklimakan Desert[J]. *Journal of Desert Research*. (2018)

[25] Zhou CL, Yang XH, Zhong XJ, Yang F, Qiu HM (2017). Dust Weather in Hinterland of the Taklamakan Desert. Arid Zone Research.

[26] Jin LL (2017). Microclimate over the Center and Edge Areas of the Artificial Shelter Forest Land in Taklimakan Desert. Journal of Desert Research.

[27] Li HQ (2016). Estimating Surface Broadband Emissivity of the Taklimakan Desert With FTIR and MODIS Data. Spectroscopy and Spectral Analysis.

[28] Wan Z (2014). New refinements and validation of the MODIS Land-Surface Temperature/Emissivity products. Remote Sensing of Environment.

[29] Yao YJ, Qin QM, Zhao SH, Yuan WL (2011). Retrieval of soil moisture based on MODIS shortwave infrared spectral feature. Journal of Infrared & Millimeter Waves.

[30] Zhou L (2003). Relations between albedos and emissivities from MODIS and ASTER data over North African Desert. Geophysical Research Letters.

[31] Cheng, J. & Liang, S. A New Algorithm for Estimating Global Bare Soil Broadband Emissivity using MODIS Albedo. *IEEE Transactions on Geoscience and Remote Sensing*., doi:10.1175/1520-0442015<3123:TLSCOT>2.0.CO;2. (2012)

[32] Ren H, Liang S, Yan G, Cheng J (2012). Empirical Algorithms to Map Global Broadband Emissivities Over Vegetated Surfaces[J]. IEEE Transactions on Geoscience & Remote Sensing.

[33] Cheng J, Liang S, Weng F, Li X (2010). Comparison of Radiative Transfer Models for Simulating Snow Surface Thermal Infrared Emissivity. IEEE Journal of Selected Topics in Applied Earth Observations & Remote Sensing.

[34] Cheng J, Liang S (2013). Estimating global land surface broadband thermal-infrared emissivity using advanced very high resolution radiometer optical data. International Journal of Digital Earth.

[35] Anonymous. Global Land Surface Characteristic Parameters (GLASS) Products [M]// Global Land Surface Feature Parameters (GLASS) products. (2014)

[36] Wilber, A. C., Kratz, D. P. & Gupta, S. K. Surface Emissivity Maps for Use in Satellite Retrievals of Longwave Radiation [M]. *NASA Langley Technical Report Server*. (1999)

[37] Wan Z, Dozier J (1996). A generalized split-window algorithm for retrieving land-surface temperature from space. IEEE Transactions on Geoscience & Remote Sensing.

